# Letter to the editor for “Update of the human and mouse Fanconi anemia genes”

**DOI:** 10.1186/s40246-016-0081-3

**Published:** 2016-07-04

**Authors:** Daniel W. Nebert, Hongbin Dong, Elspeth A. Bruford, David C. Thompson, Hans Joenje, Vasilis Vasiliou

**Affiliations:** Department of Pediatrics and Molecular Developmental Biology, Division of Human Genetics, Children’s Hospital Medical Center, Cincinnati, OH 45229-2899 USA; Department of Environmental Health and Center for Environmental Genetics, University Cincinnati Medical Center, P.O. Box 0056, Cincinnati, OH 45267-0056 USA; Department of Environmental Health Sciences, Yale School of Public Health, 60 College St., New Haven, CT 06250 USA; HUGO Gene Nomenclature Committee (HGNC), European Bioinformatics Institute-European Molecular Biology Laboratory (EMBL-EBI), Hinxton, CB10 1SD, UK; Department of Clinical Practice, University of Colorado Denver, Aurora, CO 80045 USA; Department of Clinical Genetics and the Cancer Center, Amsterdam/VUmc Institute for Cancer and Immunology, VU University Medical Center, NL-1081 BT, Amsterdam, The Netherlands

After our review of the “Update of the human and mouse Fanconi anemia genes” had been published [1], a report appeared [2] that we believe contains additional important and clarifying information relevant to our discussion of the “Fanconi anemia pathway” and cross-talk with other DNA-repair pathways.

**Fig. 1:**
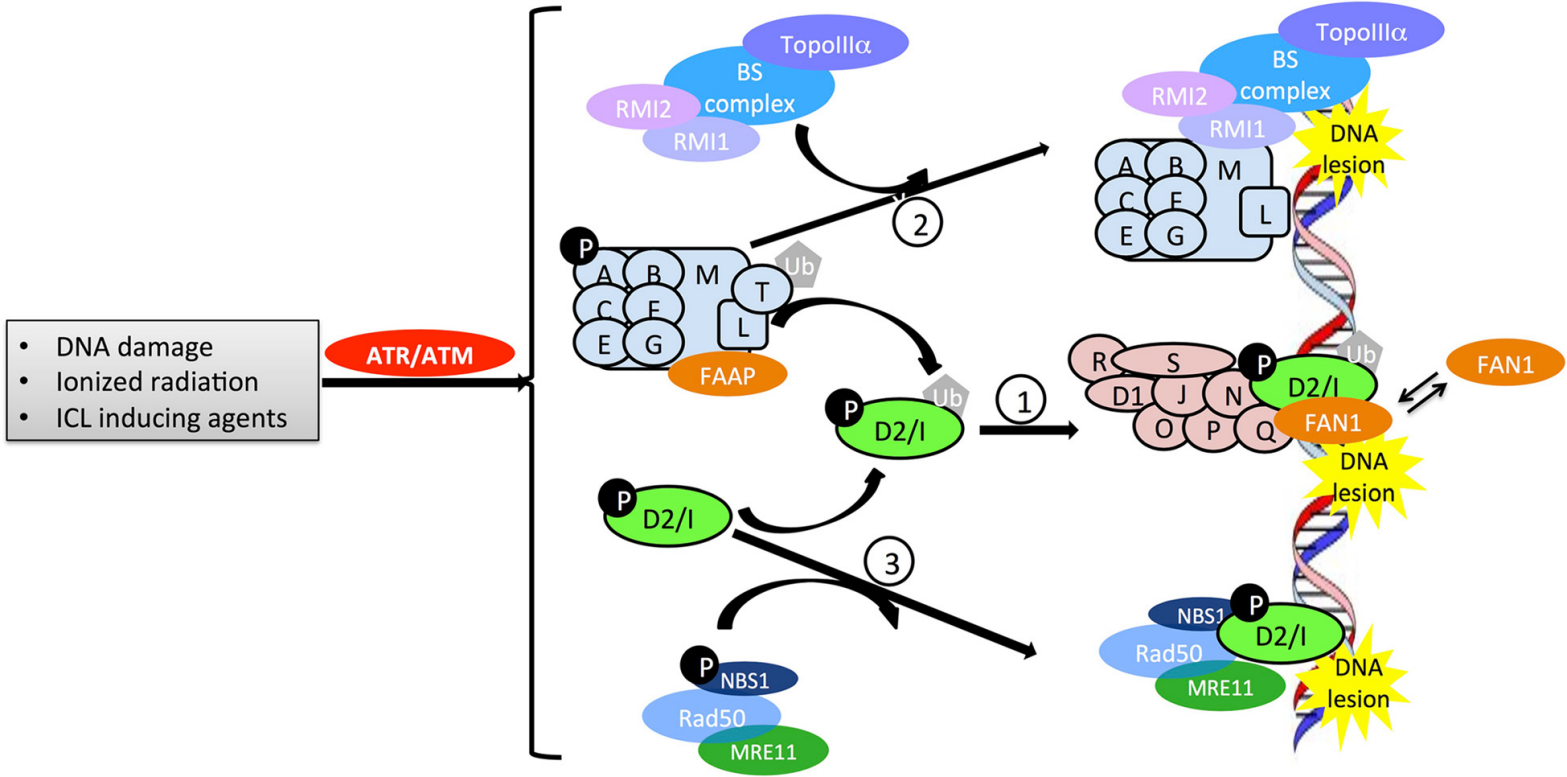
The revised diagram

In Fig. 1, which is a modified version of the Figure 2 cartoon in our recent review [1], we had shown that, in response to upstream DNA damage signaling (such as phosphorylation by ATR/ATM) in the “FA/BRCA pathway,” the FA core complex comprises at least 11 proteins. These include FANCA (A), FANCB (B), FANCC (C), FANCE (E), FANCF (F), FANCG (G), FANCM (M), and FANCL (L) proteins, plus three FAAP proteins (FAAP20, FAAP24, and FAAP100). This core complex binds UBE2T (T) by way of FANCL**;** the resultant complex then activates FANCD2/I dimers by means of mono-ubiquitination, and we had written that this is an essential prerequisite for repairing DNA interstrand cross-links.

The activated FANCD2/I (D2/I) complex then translocates to DNA damage sites and recruits downstream FA effector proteins. These include BRCA1 (S), BRCA2 (D1), RAD51 (R), BRIP1 (J), PALB2 (N), RAD51C (O), SLX4 (P), and ERCC4 (Q), plus other DNA-repair molecules (including FA-associated nuclease-1 (*FAN1*)), as illustrated as the orange ellipse at the far right in our Fig. 1 diagram [1]. This large complex then migrates to the site of the lesion to repair the DNA damage.

It had been presumed that FAN1, also required for interstrand cross-link repair, is recruited by ubiquitinated FANCD2/I (light green ellipse in Fig. 1) [1]. However, Lachaud and coworkers, using FAN1 nuclease-deficient mice, recently demonstrated [2] that recruitment of FAN1 by ubiquitinated FANCD2/I is not essential for interstrand cross-link repair. As an alternative, FAN1 recruitment and activity restrain DNA replication fork progression. This restraint, in turn, holds in check any chromosomal abnormalities from occurring, when the DNA replication forks stall. And this pause can happen––even in the absence of interstrand cross-links.

Consequently, recruitment of FAN1 by ubiquitinated FANCD2 may be regarded as facilitating the processing of stalled forks during DNA replication. Although this checkpoint process is essential for genome stability and improved overall well-being of the cell, it might better be described as not absolutely necessary. Therefore, a slightly revised *figure* is proposed here, in which FAN1 can be either bound or not bound from the remainder of this complex (comprising D2/I, S, D1, R, J, N, O, P, Q, and other DNA repair molecules). This *revised diagram* now takes into account the latest data published by Lachaud et al. [2].

Future experiments using other knockout mice will clarify this pathway further. Given the recent advances with CRISPR/*Cas9* methodology, knockout mouse lines––or cells in culture––can be efficiently created for all components shown in the *figure* herein, and then all permutations can be examined, one-by-one.
